# Bacterial diversity of middle ear cholesteatoma by 16S rRNA gene sequencing in China

**DOI:** 10.1007/s10142-023-01068-2

**Published:** 2023-04-27

**Authors:** Qiulin Liang, Ruiqing Long, Shuling Li, Chaowu Jiang, Jingyu Gao, Sheng Cheng, Zhuohui Liu, Biao Ruan

**Affiliations:** Department of Otolaryngology, The First Affiliated Hospital of Kunming Medical University, Kunming, 650032 Yunnan China

**Keywords:** Chronic otitis media, Middle ear cholesteatoma, Bacterial diversity, 16S rRNA, sequencing

## Abstract

In this study, the bacterial diversity of acquired middle ear cholesteatoma (MEC) was evaluated to reveal its pathogenesis and provides a guide for the use of antibiotics. Twenty-nine cases of acquired MEC and eight cases of healthy middle ears undergoing cochlear implantation (CI) were evaluated. Full-length 16S rRNA gene sequencing was performed to profile the bacterial communities in lesions and healthy tissues of the middle ear. ACE (*P* = 0.043) and Chao1 (*P* = 0.039) indices showed significant differences in alpha diversity (*P* < 0.05). Analysis of PERMANOVA/Anosim using the Bray–Curtis distance matrix results suggested that the between-group differences were greater than the within-group differences (*R* = 0.238, *P* < 0.05, *R*^2^ = 0.066, *P* < 0.05). Bacterial community analysis revealed that *Alphaproteobacteria* at the class level and *Caulobacterales* and *Sphingomonadales* at the order level were significantly different (*P* < 0.05). In the LefSe (Linear discriminant analysis effect size) analysis, *Porphyromonas bennonis* was elevated, and *Bryum argenteum* and unclassified *Cyanobacteriales* were reduced at the species level in MEC (*P* < 0.05). Fifteen metabolic pathways were found to be significantly different between the two groups by analysing the abundance of metabolic pathways in level 2 of the Kyoto Encyclopaedia of Genes and Genomes (KEGG). Seven and eight metabolic pathways were significantly elevated in the MEC and control groups, respectively (*P* < 0.05). The role of bacteria in the pathogenesis of acquired MEC was further refined through analysis of metabolic pathways. These findings indicate that the acquired MEC and healthy middle ear contain more diverse microbial communities than previously thought.

**Supplementary Information:**

The online version contains supplementary material available at 10.1007/s10142-023-01068-2.

## Introduction

Cholesteatoma is a cystic pseudotumour comprising keratinised squamous epithelia, which produces keratin masses (Beláková et al. 2019), and cholesteatoma contains congenital cholesteatoma and acquired cholesteatoma. Acquired middle ear cholesteatoma (MEC) is the most severe disease of the middle ear worldwide. It is characterised by otorrhea and hearing loss and may lead to severe intra-and extracranial complications (Yamamoto-Fukuda and Akiyama, 2020; Luntz and Barzilai, 2021). Its treatment is always surgical, and the operation focuses on removing the cholesteatoma and repairing damaged structures, such as the ossicular chain (Callesen et al. 2021).

At present, the pathogenesis of acquired MEC remains unclear. Most acquired MEC cases are secondary to chronic suppurative otitis media, and microorganisms play an important role in the development. Bacterial biofilms have also been found in acquired MEC, supporting the role of bacterial infection in its development (Saylam et al. 2010; Khomtchouk et al. 2021). Ear infection and Eustachian tube (ET) dysfunction are likely to trigger the development of acquired cholesteatoma. Chronic inflammation also seems to play a fundamental role in multiple etiopathogenic mechanisms of acquired MEC (Persaud et al. 2007). Therefore, there is a need to elucidate its pathogenesis of acquired MEC and the use of antibiotics. The study of microorganisms is often based on culturing techniques, in which only some of the dominant bacteria can be successfully detected and numerous relevant bacteria are not identified. Nevertheless, because several human microorganisms are non-culturable or extremely difficult to culture, molecular tools can provide a better understanding of the bacterial community composition in a given sample. Large-scale sequencing enables the characterisation of entire bacterial populations via the parallel analysis of 16S rRNA genes from the entire population. Theoretically, its unbiased nature allows for the detection of almost all bacteria present in a sample (Sillanpää et al. 2017). In this study, the full-length 16S rRNA gene sequenced by third-generation sequencing (TGS) was used to analyse the bacterial spectrum of acquired MEC, the main objective being to facilitate a better understanding of the aetiology and treatment of this disease.

## Methods

### Patient information

Twenty-nine patients diagnosed with acquired MEC that underwent surgical treatment in the Otolaryngology Department of the First Affiliated Hospital of Kunming Medical University (B1–B29, MEC group, Supplementary Table 1) were enrolled in the study. Eight patients who received cochlear implantation (CI) were included in the control group (C1–C8, control group). Patients who received antimicrobial treatment (either systemic or ototopical) within 2 weeks of surgery and those who were immunodeficient were excluded. All study procedures were approved by the Ethics Committee of the First Affiliated Hospital of Kunming Medical University and were performed only after obtaining written informed consent from the adult individual or the child’s parents (if under 16).

Middle ear lesion tissues from the MEC group and mastoid mucosa from the control group were collected intraoperatively after opening the tympanic sinus and the mastoid cavity, respectively. Pathological tissues were immediately stored in liquid nitrogen until batch processing.

### 16S rRNA sequencing

DNA was isolated from the specimens and processed for microbiome analysis by 16S rRNA gene sequencing. Genomic DNA was extracted using a PowerSoil^®^ DNA Isolation Kit, according to the manufacturer’s instructions. The extracted total DNA was subjected to PCR using specific primers with barcodes synthesised based on the full-length primer sequences (27F: 5′-AGRGTTTGATYNTGGCTCAG-3′, 1492R: 5′- TASGGHTACCTTGTTASGACTT-3′). The PCR conditions were as follows: 95 °C for 5 min, 25/30 cycles of 95 °C for 30 s, 50 °C for 30 s, and 72 °C for 60 s/1 kb, and lastly 72 °C for 7 min). The PCR products were purified, quantified, and homogenised to form a sequencing library (SMRT Bell). The data from PacBio Sequel were exported in bam format, and circular consensus sequencing (CCS) files were exported using SMRT Link v8.0. Data from different samples were identified according to their barcode sequences and converted to FastQ format.

After exporting the PacBio downlink data to a CCS file (the CCS sequence was obtained using the SMRT Link v8.0 provided by PacBio), the data were pre-processed in three main steps: (i) CCS identification: raw CCS sequence data obtained by barcode identification of CCS using lima v1.7.0 software; (ii) CCS filtering, identification, and removal of primer sequences; and (iii) length filtering using Cutadapt 1.9.1 software to obtain clean CCS sequences that did not contain primer sequences. UCHIME v8.1 software was used to identify and remove chimeric sequences and obtain effective CCS sequences.

### Bioinformatic analysis

Unique sequences were clustered using USEARCH v10.0 into operational taxonomic units (OTUs) at a threshold of 97% 16S rRNA gene sequence similarity. Using SILVA (release 132, http://www.arb-silva.de) as the reference database, the taxonomic annotation of the feature sequences was performed using a simple Bayesian classifier combined with a comparison method to obtain the taxonomic information of the species corresponding to each feature. The community composition of each sample was determined at each level (phylum, class, order, family, genus, and species). A table of species abundance at different taxonomic levels was generated using QIIME software, and the community structure of the samples at each taxonomic level was plotted using *R*.

Mothur v1.30 was used to analyse the alpha diversity of each sample within the two groups including Chao1 richness estimator, ACE richness estimator, Shannon diversity index, and Simpson diversity index. These indicators were primarily a response to species richness and diversity in the community.

The “vegan” package in *R* was used for PERMANOVA/Anosim. PERMANOVA/Anosim analysis was used to assess the differences between all samples between the groups. The *R*^2^ obtained from the PERMANOVA analysis indicated the degree of explanation by subgroup for the differences in samples. Anosim analysis yielded an *R* value closer to 1, indicating that the between-group differences were greater than the within-group differences, whereas a smaller *R* value indicated that there were no significant between-group differences.

Linear discriminant analysis effect size (LefSe) (http://huttenhower.sph.harvard.edu/lefse/) was used to determine the magnitude of the effect of species abundance on variation in each group, thereby identifying biomarkers that differed significantly in abundance between the two groups. An LDA (linear discriminant analysis) score of > 4 was considered significantly different.

Based on the abundance and variation of each species in each sample, Spearman’s rank correlation analysis was performed, and data with correlations greater than 0.1 and *P* < 0.05 were screened to construct a correlation network. Based on the analysis of the network diagrams, the co-occurrence of species in the environmental samples can be obtained, and the interaction of species in the same environment and important pattern information can be obtained to explain the formation mechanism of phenotypic differences between samples. The species correlation network map was drawn based on python.

PICRUSt2 (Phylogenetic Investigation of Communities by Reconstruction of Unobserved States, 2019.10) was used to annotate the functional sequences to be predicted with the species in the phylogenetic tree available in the software, and the Integrated Microbial Genomes data was used to output functional information to infer the functional gene composition of the samples and analyse the functional differences between the control and MEC groups. Kyoto Encyclopaedia of Genes and Genomes (KEGG) analysis was used to evaluate the differences and changes in the metabolic pathways of the functional genes of the microbial community between the two groups.

### Statistical analysis

Analysis of PERMANOVA/Anosim between the two groups was performed using the Bray–Curtis distance matrix. Wilcoxon rank-sum tests were performed to estimate significant differences between variables using the GraphPad Prism software. *P* <0.05 was considered statistically significant for all analyses.

## Results

**Fig. 1:**
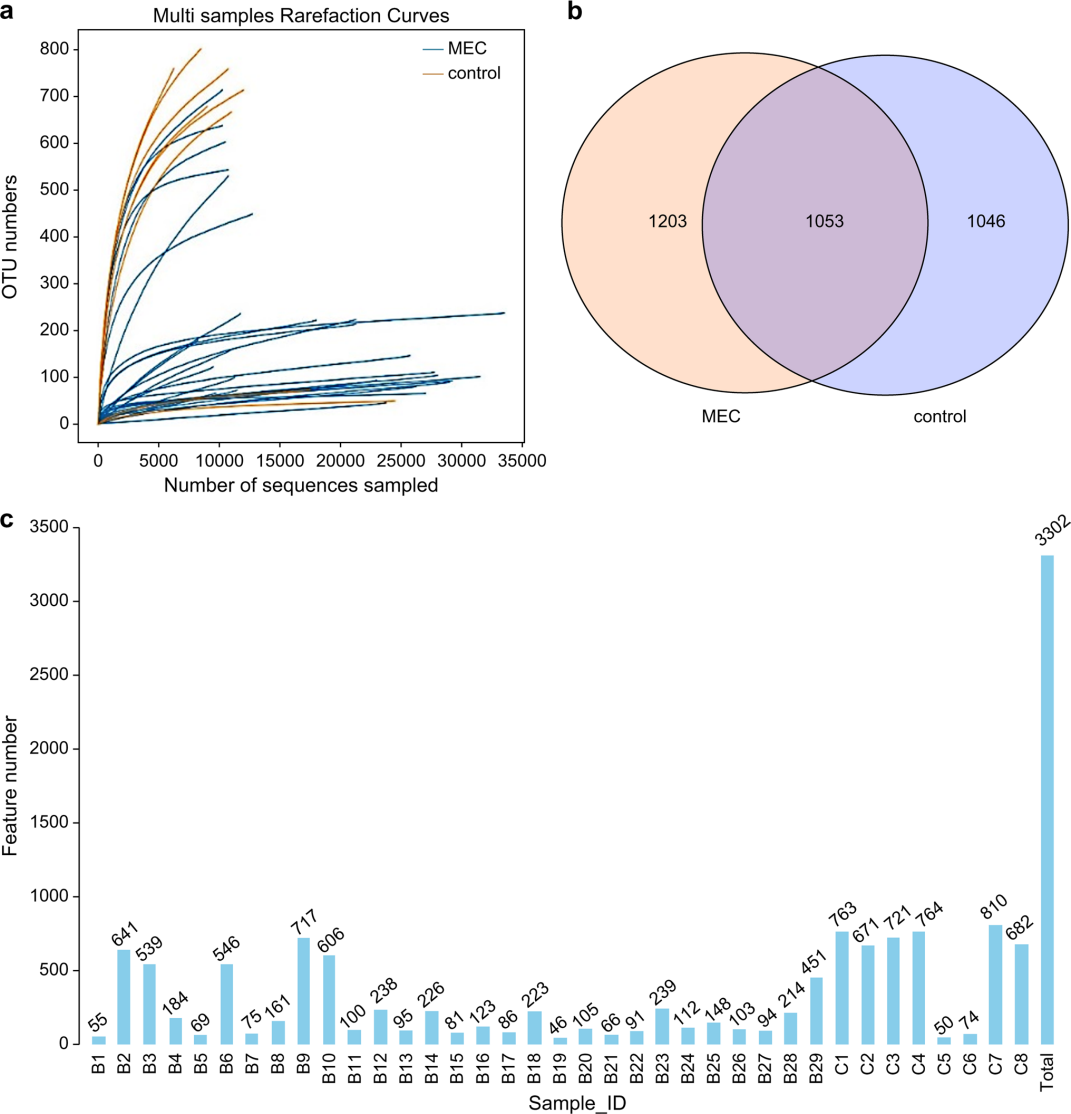
Rarefaction curves for each sample in the MEC and control groups. (**a**) Venn diagram of the OTUs from the MEC and control groups. (**b**) Number of OTUs in each sample. (**c**) Middle ear cholesteatoma is indicated by “MEC” and heathy middle ear by “control”

A total of 775,000 CCS reads were obtained by sequencing using the PacBio platform. A total of 751,429 effective CCS remained after filtering using Cutadapt 1.9.1 and chimaeras were removed using UCHIMEv8.1 (Table 1). The sequencing depth was considered sufficient for further research from the rarefaction curves, which had a distinct levelling-off phase for all samples (Fig. 1a). The total number of detected OTUs was 3302, comprising 1203 unique OTUs detected in the MEC, 1046 unique OTUs in the control group, and 1053 OTUs that were common in the Venn diagram between the two groups (Fig. 1b, c).

**Table 1:** Sample sequencing data processing statistics

Sample ID	Raw CCS	Clean CCS	Effective CCS	AvgLen (bp)	Effective (%)
B1	13,097	12,973	12,145	1464	92.73
B2	13,013	12,982	12,385	1450	95.17
B3	12,948	12,941	12,855	1472	99.28
B4	9876	9866	9807	1478	99.3
B5	11,246	11,183	11,093	1463	98.64
B6	12,954	12,934	12,490	1452	96.42
B7	12,327	12,309	12,302	1479	99.8
B8	13,045	13,039	13,032	1478	99.9
B9	12,966	12,911	12,229	1456	94.32
B10	13,009	12,978	12,613	1453	96.96
B11	13,020	13,017	12,941	1468	99.39
B12	12,920	12,917	12,893	1474	99.79
B13	26,111	25,948	25,804	1456	98.82
B14	24,079	23,934	23,574	1472	97.9
B15	28,826	28,469	28,344	1461	98.33
B16	12,964	12,804	12,709	1466	98.03
B17	27,498	27,426	27,185	1460	98.86
B18	24,400	24,281	21,596	1458	88.51
B19	24,735	24,712	24,684	1456	99.79
B20	30,738	30,613	30,350	1451	98.74
B21	32,945	32,754	31,770	1461	96.43
B22	32,149	32,095	31,935	1468	99.33
B23	37,861	37,638	37,280	1456	98.47
B24	30,282	30,160	29,933	1461	98.85
B25	28,303	28,237	28,118	1470	99.35
B26	34,173	34,001	33,722	1454	98.68
B27	33,715	33,528	33,218	1456	98.53
B28	29,427	28,919	27,192	1461	92.4
B29	19,902	19,866	17,784	1472	89.36
C1	13,086	13,058	12,878	1454	98.41
C2	12,966	12,934	12,498	1463	96.39
C3	26,021	25,978	23,333	1444	89.67
C4	12,526	12,505	11,903	1444	95.03
C5	26,940	26,905	26,845	1476	99.65
C6	19,430	19,406	19,387	1474	99.78
C7	17,459	17,432	16,285	1445	93.28
C8	18,043	18,021	16,317	1443	90.43

*AvgLen*, average length

### Diversity analysis

**Fig. 2:**
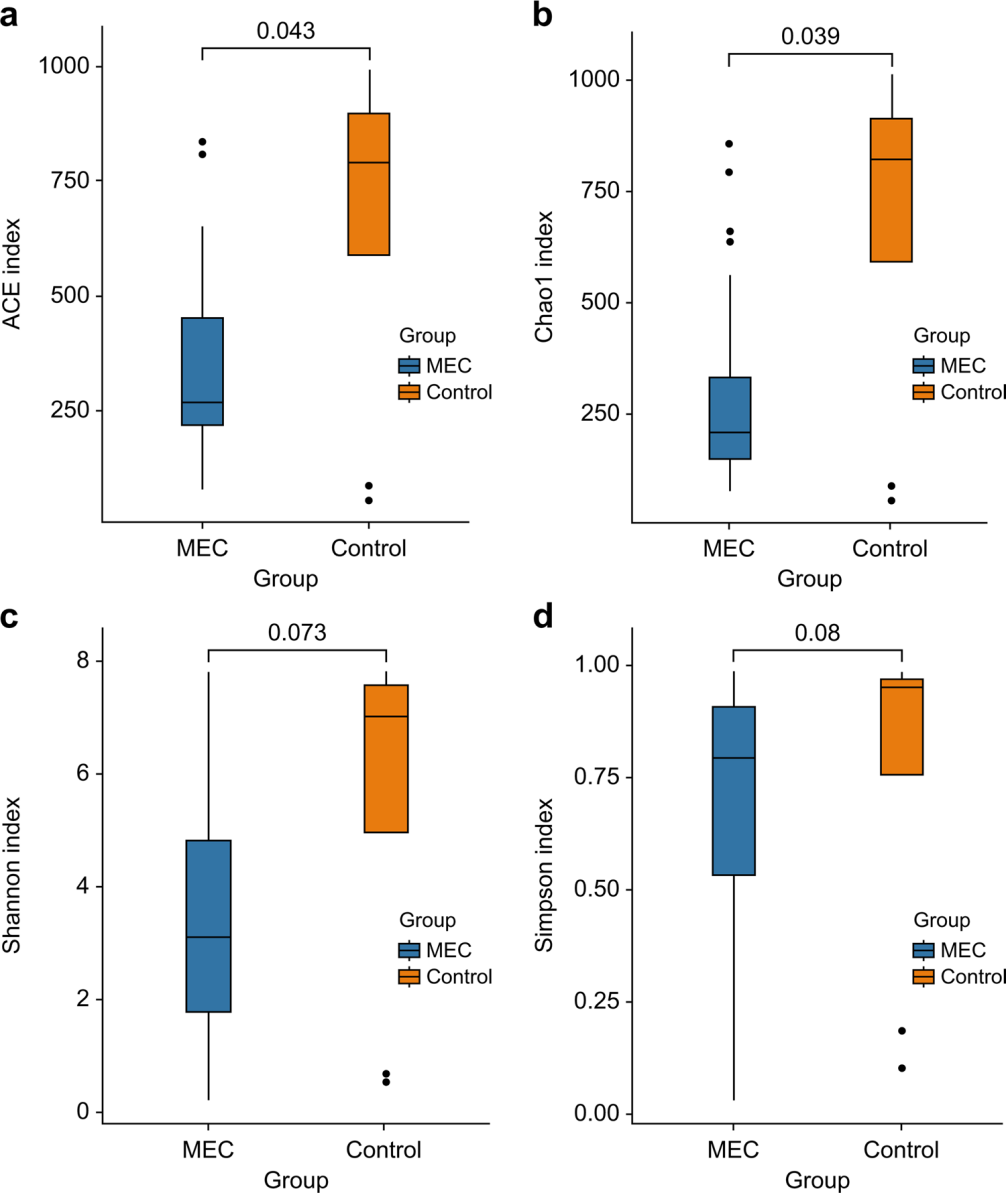
Alpha diversity in the MEC and control groups. (**a**) ACE richness estimator. (**b**) Chao1 richness estimator. (**c**) Shannon diversity index. (**d**) Simpson diversity index. Middle ear cholesteatoma is indicated by “MEC” and heathy middle ear by “control” on the *x*-axis of each plot

**Fig. 3:**
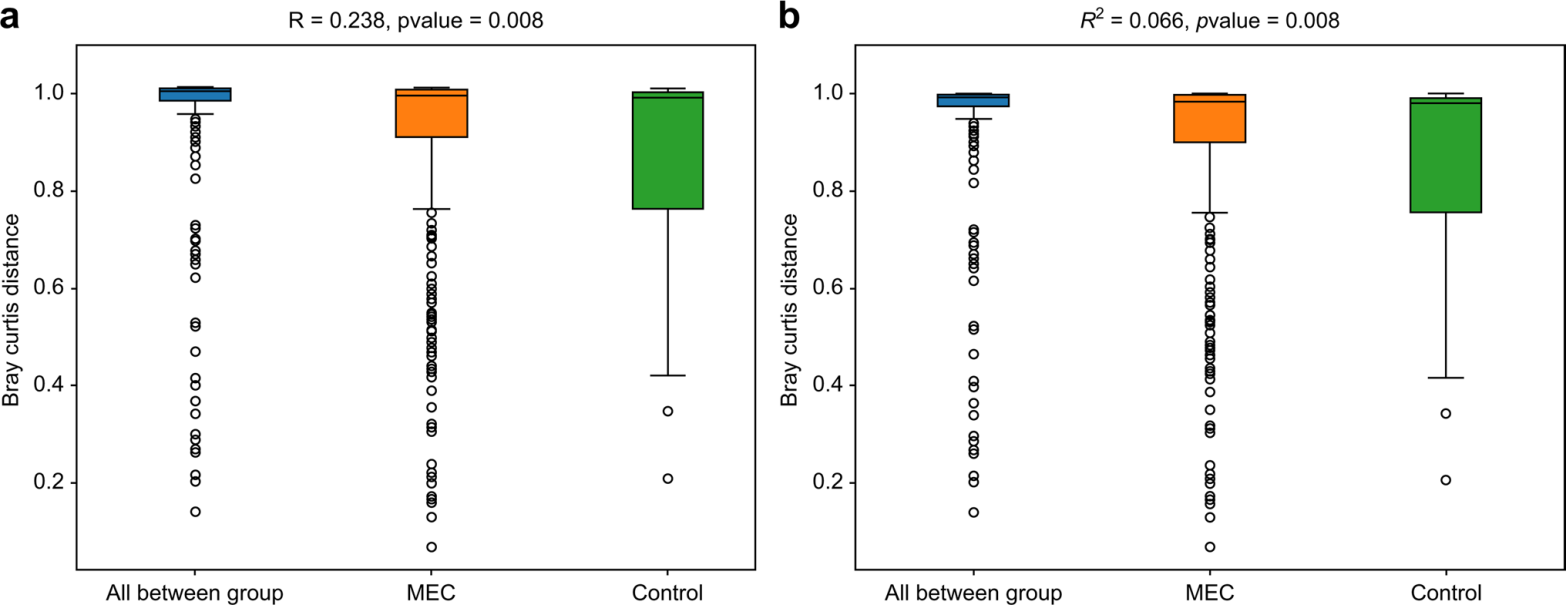
PERMANOVA/Anosim analysis of the MEC and control groups based on the Bray–Curtis distance matrix. Middle ear cholesteatoma is indicated by “MEC” and heathy middle ear by “control” on the *x*-axis of each plot

The ACE (*P* = 0.043) and Chao1 (*P* = 0.039) indices showed significant differences in alpha diversity (*P* < 0.05). In contrast, the Shannon (*P* = 0.073) and Simpson (*P* = 0.08) indices showed no differences in alpha diversity (*P* > 0.05) (Fig. 2). This suggests that the control group had higher species richness than the MEC group. Analysis of PERMANOVA/Anosim using the Bray–Curtis distance matrix results suggested that the between-group differences were greater than the within-group differences (*R* = 0.238, *P* = 0.008; *R*^2^ = 0.066, *P* = 0.008) (Fig. 3).

### Taxonomic analysis of species

**Fig. 4:**
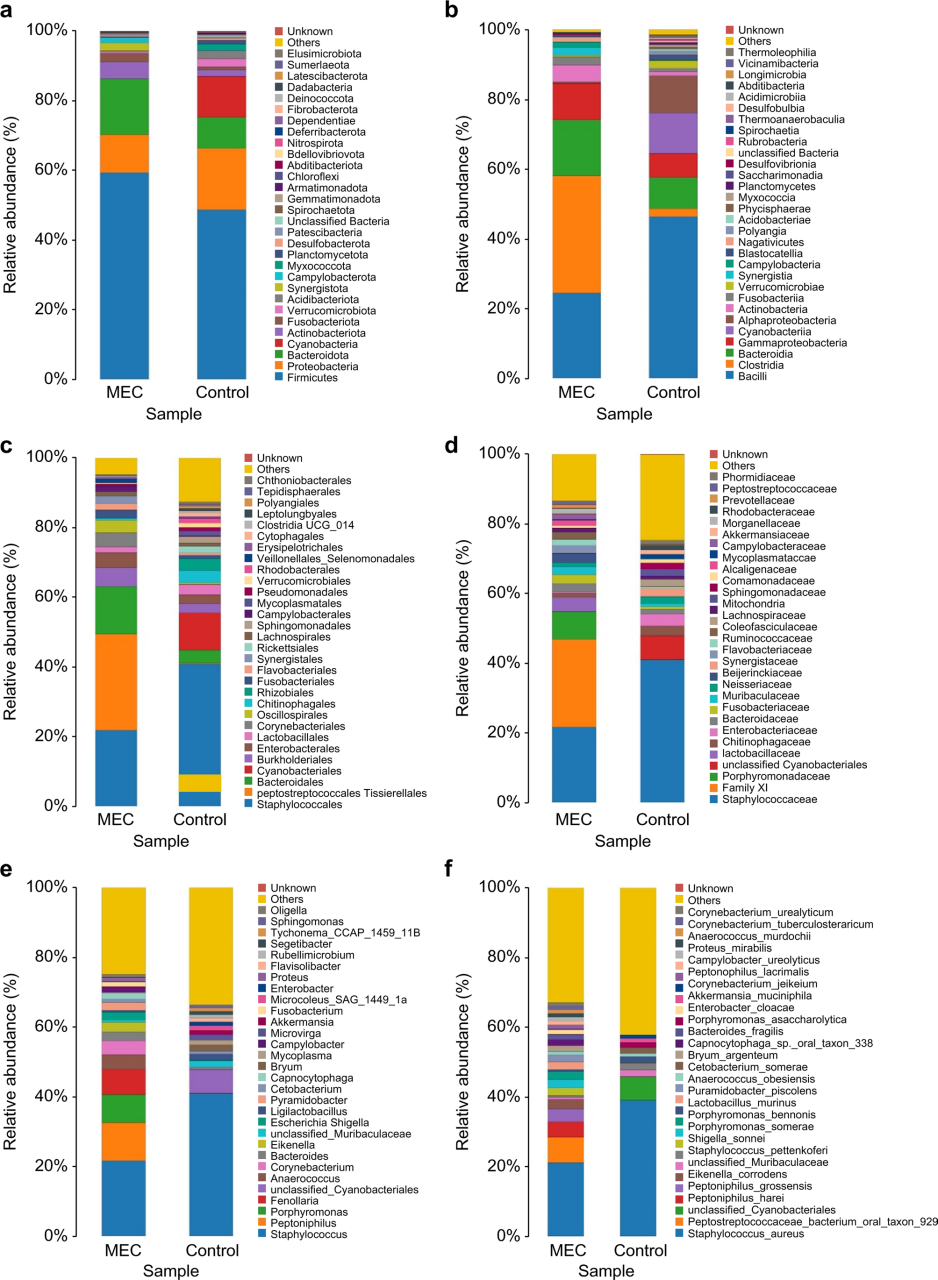
16S rRNA gene-based bacterial community compositions of the MEC and control groups. Horizontal axes indicate the different samples, and the vertical axes indicate the different abundances of species at different taxonomic levels. **a** Phylum. **b** Class. **c** Order. **d** Family. **e** Genus. **f** Species. Different colours on the right-hand side of the diagram indicate the different species classifications at different taxonomic levels. Middle ear cholesteatoma is indicated by “MEC” and heathy middle ear by “control” on the *x*-axis of each plot

At the phylum level, the highest abundance in the MEC and control groups was *Firmicutes* (59.36% vs. 48.75%, respectively) (Fig. 4a). At the class level, the highest abundance in the MEC group was *Clostridia* (33.70%), and that in the control group was *Bacilli* (46.35%) (Fig. 4b). At the order level, the highest abundance in the MEC group was *Peptostreptococcales_Tissierellales* (27.59%), and that in the control group was *Staphylococcales* (40.95%) (Fig. 4c). At the family level, the highest abundance in the MEC group was *Family_XI* (25.11%), and that in the control group was *Staphylococcaceae* (40.95%) (Fig. 4d). At the genus level, the highest abundance in the MEC and control groups was *Staphylococcus* (21.63% vs. 40.95%, respectively) (Fig. 4e). At the species level, the highest abundance in the MEC and control groups was *Staphylococcus aureus* (21.14% vs. 39.01%, respectively) (Fig. 4f). *Alphaproteobacteria* at the class level and *Caulobacterales* and *Sphingomonadales* at the order level were significantly different between the two groups (*P* < 0.05) (Table 2).

**Table 2:** Differentially abundant bacteria at different taxonomic levels between the two groups

Phylogenetic level	MEC (%)	Control group (%)	*P* value	*P*-corrected
Class				
Alphaproteobacteria	0.853	14.530	0.001	0.047
Order				
Caulobacterales	0.097	0.601	<0.001	0.037
Sphingomonadales	0.107	2.364	0.001	0.049

*MEC*, middle ear cholesteatoma

**Fig. 5:**
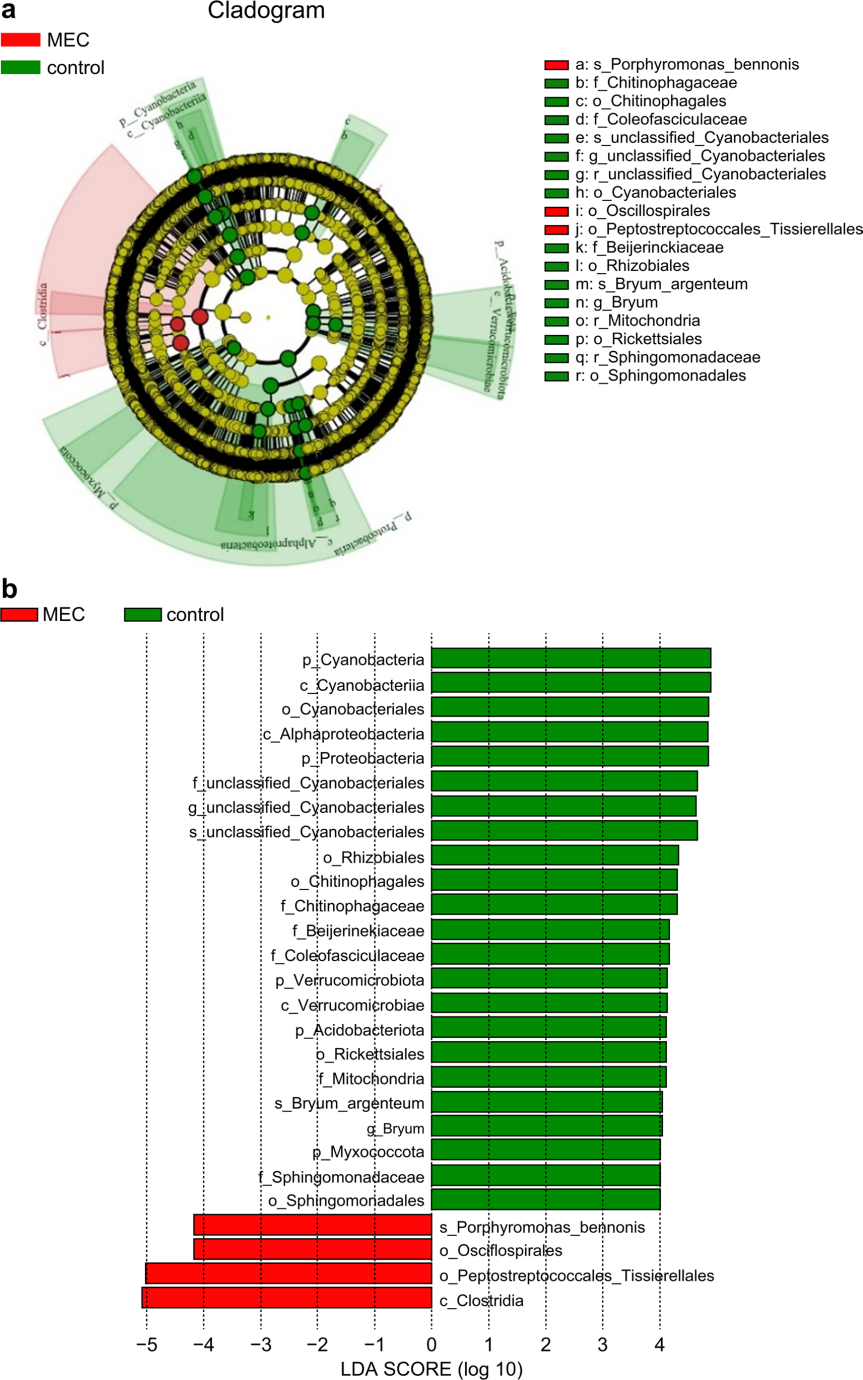
(**a**) Branching evolution chart of the MEC and control groups in the LefSe analysis. The circles radiating from the inside out represent taxonomic levels from phylum to species; the small circles at different taxonomic levels represent a grouping of taxa; the diameter of the small circles represents the relative abundance size of the species; and the coloured nodes indicate species that play an important role in the same colour grouping. Different colours on the right-hand side of the diagram indicate the different species classifications at different taxonomic levels. (**b**) Bar chart of the MEC and control groups based on the LefSe analysis (LDA score > 4). Middle ear cholesteatoma is indicated by “MEC” and heathy middle ear by “control” in each plot

In the LefSe analysis, *Clostridia* at the class level, *Oscillospirales* and *Peptostreptococcales_Tissierellales* at the order level were abundant in the MEC group. *Porphyromonas bennonis* was elevated, and *Bryum argenteum* and *unclassified_Cyanobacteriales* were reduced at the species level in the MEC group (LDA score > 4, *P* < 0.05) (Fig. 5).

**Fig. 6:**
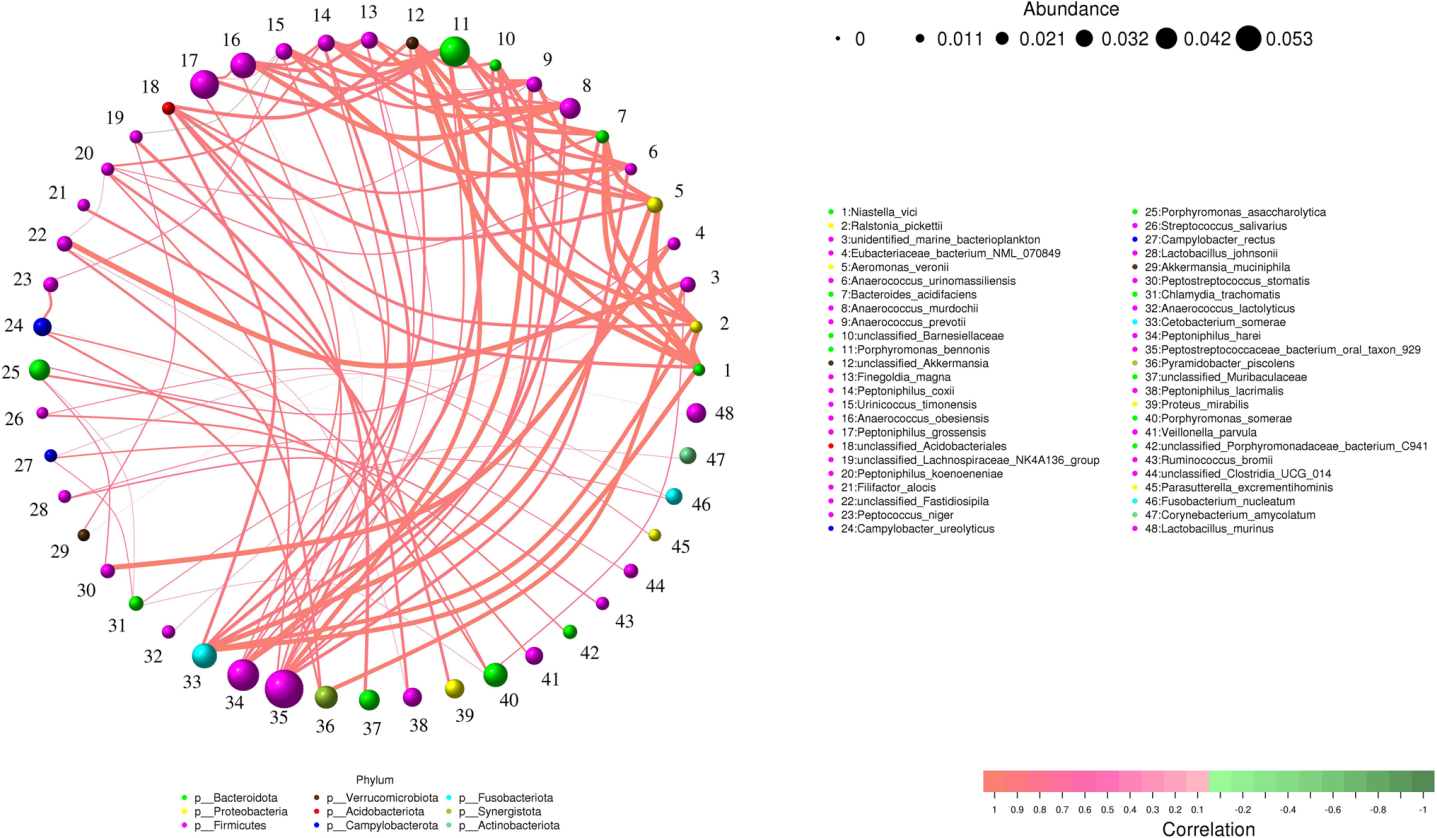
The species correlation network diagram in MEC group. Each circle represents a species; the size of the circle represents the abundance of the species; the different colours represent different phylum; the red lines represent positive correlations; the green lines represent negative correlations; the right side of the graph shows the specific annotations of the different species (correlation factor > 0.1, *P* < 0.05). Middle ear cholesteatoma is indicated by “MEC”

Top 80 species abundances in the MEC group were used for correlation network analysis, and a total of 48 species showed the strongest correlations, with 99 positive correlation lines and one negative correlation line. *Niastella_vici*, *Ralstonia_pickettii* and *Aeromonas_veronii* were the most strongly correlated (Fig. 6).

### KEGG metabolic pathway analysis

The abundance of the metabolic pathways at level 2 based on the KEGG database was analysed to compare the differences in metabolism between the two groups of bacteria. By analysing the metabolic pathway at level 2 of the KEGG metabolic pathway, 45 metabolic pathways were annotated, and 15 metabolic pathways were significantly different between the two groups. Seven metabolic pathways were significantly elevated in MEC, mainly including nucleotide metabolism, translation, replication, repair, folding, sorting, and degradation (*P* < 0.05). Eight metabolic pathways were significantly elevated in the control group, mainly including amino acid metabolism, xenobiotic biodegradation and metabolism, neurodegenerative diseases, and drug resistance: antineoplastic (*P* < 0.05) (Table 3).

**Table 3:** Differential enrichment of KEGG metabolic pathway analysis

KEGG metabolic pathway	MEC (%)	Control group (%)	*P*-corrected
Folding, sorting, and degradation	1.607	1.471	0.001
Immune diseases	0.049	0.040	0.001
Nucleotide metabolism	4.117	3.323	<0.001
Replication and repair	3.286	2.546	<0.001
Signaling molecules and interaction	0.046	0.035	0.001
Transcription	0.188	0.153	0.005
Translation	3.834	2.995	<0.001
Amino acid metabolism	6.434	7.046	0.004
Cardiovascular diseases	0.005	0.024	0.038
Drug resistance (antineoplastic)	0.022	0.098	0.048
Infectious diseases (parasitic)	0.044	0.071	0.001
Infectious diseases (viral)	0.013	0.066	0.036
Neurodegenerative diseases	0.225	0.333	0.020
Substance dependence	0.005	0.026	0.047
Xenobiotics biodegradation and metabolism	1.114	1.472	0.001

*MEC*, middle ear cholesteatoma

## Discussion

Acquired MEC is a common condition in the field of otolaryngology. Therefore, the subjects included in this study were all acquired MEC. Bacterial infections play an important role in the onset and development of acquired MEC, and microbial communities play an indispensable role in all ecosystems, particularly living organisms (Lemanceau et al. 2017; Simon et al. 2019). Thus, an understanding of the bacterial spectrum of acquired MEC has fundamental applications in the use of drugs, especially antibiotics, for its treatment.

Although several studies have been conducted on the bacterial profile of acquired MEC, all have used culture-based methods and showed few dominant species, providing an incomplete picture of the MEC bacterial profile (Xu et al. 2020; Xu et al. 2021). In this study, the full-length 16S rRNA gene sequenced by TGS was used to analyse the bacterial spectrum of acquired MEC. Compared with the full-length 16S rRNA gene sequenced by TGS, the V3 and V4 regions of the 16S rRNA gene sequenced using next-generation sequencing (NGS) were found to have a lower resolution, and may incorrectly estimate the composition of microbial communities (Yang et al. 2021). In particular, the identification of bacteria using NGS is limited to the genus level because of the current specificity of PCR primers used in 16S rRNA gene sequencing (Minami et al. 2017) Therefore, it is advantageous to select the 16S rRNA gene sequenced by TGS to study the MEC bacterial profile. In contrast, cultivation-independent molecular techniques can provide a more accurate assessment of microbial communities growing on mucosa and lesion tissues (Neeff et al. 2016). As molecular diagnostic techniques become part of routine testing, clinicians must learn how to correctly use them under the correct clinical settings to harness the full spectrum of their functionality.

In the present study, patients with a healthy middle ear who underwent CI were selected as the control group. It is a long-held belief that the middle ear of a healthy individual is sterile (Westerberg et al. 2009; Chang et al., 2011). However, the results of this study showed an abundant bacterial community in healthy middle ears, which had a higher species richness than that of MEC. The ACE and Chao1 indices showed significant differences in alpha diversity (*P* < 0.05). Bacterial community analysis at all levels, including phylum, class, order, family, genus, and species, revealed common bacteria between the two groups. A total of 1053 OTUs, which were common in the two groups, indicated the presence of similar bacteria between the MEC and healthy middle ears. However, *Staphylococcus aureus* remained the predominant bacterium in both groups. This is similar to the results of many previous studies (Mittal et al. 2015; Xu et al. 2021). Although there were similarities in some bacteria between the two groups, the abundance of bacteria differed. This was also an indirect indication that acquired MEC may arise from the middle ear or mastoid commensal bacteria alone under certain conditions leading to pathogenicity, such as unregulated antibiotic use and/or compromised immunity. Some resident bacteria were detected in the peripheral organs and bacteria isolated from the oral cavity, including *Porphyromonas asaccharolytica*, *Eikenella corrodens*, and *Porphyromonas* sp*.* (Karpiński, 2019; Tanaka et al. 2020; Crooks et al. 2021), further confirming that the bacteria in chronic otitis media may originate from adjacent organs (Khattak et al. 2017). Furthermore, bacteria may enter the middle ear cavity through the ET under certain conditions, leading to acquired MEC formation (Murphy and Parameswaran, 2009).

The ET is important in the regulation of middle ear pressure (Kobayashi et al. 2009). The predominant functions of the ET are ventilation, drainage, and protection against ascending infections of the middle ear (Park et al. 2013). ET dysfunction is associated with chronic suppurative otitis media and MEC (Todt et al. 2021). Low ventilation of the ET may result in lower oxygen levels in the middle ear cavity; thus, the abundance of anaerobic bacteria, including *Anaerococcus obesiensis*, *Eikenella corrodens*, *P. asaccharolytica*, and *Porphyromonas endodontalis*, was higher than that in the control group. This also showed the importance of improving the ventilation of the tympanic cavity and ET to control infection. Therefore, anaerobic bacteria should be carefully targeted when using antibiotics to treat acquired MEC; otherwise, treatment may be less effective. In contrast, *Lactobacillus murinus* and *Akkermansia muciniphila* were lower in the MEC group than that in the control group. To the best of our knowledge, these two bacteria are the most common probiotics, suggesting that dysbiosis could play a role in the development of acquired MEC, providing a theoretical basis for the use of probiotics as a therapeutic strategy in the future.

In the LefSe analysis, the abundance of *P. bennonis* increased, while that of *B. argenteum* and *unclassified Cyanobacteriales* decreased at the species level in the MEC group. *P. bennonis* is a novel anaerobic, non-spore-forming, gram-negative bacillus, indigenous to the bacterial flora in the oral cavity of humans and animals (Summanen et al. 2009). It is sometimes considered a true pathogen and is associated with human and animal infections. *P. bennonis* is the main causative agent of bacterial meningitis (Luo et al. 2021). In mixed infections, *P. bennonis* produces β-lactamase and releases it into the immediate environment to protect other penicillin-susceptible bacteria (Brook, 2004). In certain cases, this may also lead to dysbiosis in the middle ear cavity. The discovery of these biomarkers provides new directions and targets for future research on the role and mechanisms of bacterial infection in the pathogenesis of acquired MEC. Alterations in these flora could be the core flora in the development and progression of acquired MEC.

In the correlation network analysis, *Aeromonas_veronii* showed the strongest correlation with *Niastella_vici* and *Ralstonia_pickettii*, reflecting the important role of *Aeromonas_veronii* in the MEC group. The pathogenic disease of *Aeromonas veronii* in MEC is less well studied. *Aeromonas veronii* is an opportunistic pathogen of fish-human-livestock (Zhang et al. 2022). The literature shows that the pathogenic potential of *Aeromonas veronii* is considered multifactorial, and the presence of several virulence factors allows these bacteria to adhere, invade, and destroy the host cells, overcoming the immune host response (Fernández-Bravo and Figueras, 2020). Eight virulence genes related to pathogenicity including enterotoxin, lipase, elastase, quorum sensing, hemolysin, and adhesion were identified in *Aeromonas veronii* isolate (Xu et al. 2022). Direct injection of precursor vector proteins into eukaryotic host cells by *Aeromonas veronii* promotes bacterial infection of host cells or causes apoptosis of host cells (Liu et al. 2022). However, the specific pathogenic disease of *Aeromonas veronii* in MEC needs to be verified using animal models.

The analysis of the composition and differences in KEGG metabolic pathways and functional genes in microbial communities between different groups of samples is an effective means of studying the changes in metabolic function that occur in community samples in response to environmental changes. In the present study, the KEGG pathways of nucleotide metabolism, translation, replication, and repair were the most distinctive differences in the MEC group, suggesting that the proportion of bacteria in the clonal proliferative state was significantly higher in MEC. This also highlights the importance of bacteria in the pathogenesis of the disease.

In conclusion, the bacterial profile of the normal middle ear cavity mucosa was relatively rich, while that of acquired MEC was inhabited by more diverse microbial communities. The microbial composition of acquired MEC differed from that of the healthy middle ear, which was particularly reflected in the reduced abundance of some probiotic bacteria and the elevated abundance of anaerobic bacteria. These results on bacterial profiles provide insights into the microbial distribution and pathogenic mechanisms of acquired MEC, and could be used as a guide for the targeted use of antibiotics, instead of empirical antibiotics, reducing the likelihood of spreading multidrug-resistant bacteria. However, it is worth mentioning that the sample size of this study was small, and the annotated differential functions were not validated in animal models. Thus, further research will be needed to validate these results.

## Supplementary information

ESM 1 (DOCX 19 kb)

## Data Availability

The datasets generated and/or analysed during the current study are available from the corresponding author upon reasonable request.
