# Supplementary material for: Bacterial diversity of middle ear cholesteatoma by 16S rRNA gene sequencing in China
Source: Funct Integr Genomics. 2023 Apr 27;23(2):138. doi: 10.1007/s10142-023-01068-2 (PMC10140134; doi:10.1007/s10142-023-01068-2)
Supplement: Supplementary file 1 — (DOCX 19 kb) [file 10142_2023_1068_MOESM1_ESM.docx]

Supplementary Table 1 Basic information of the MEC group

| Sample ID | Age  (years) | gender | chief complaint symptoms | onset time  (years) | cholesteatoma classification | history of antibiotic use |
| --- | --- | --- | --- | --- | --- | --- |
| B1 | 14 | male | earache | 2 | acquired | Intermittent use |
| B2 | 25 | male | hearing loss | 5 | acquired | Intermittent use |
| B3 | 47 | male | earache | 10 | acquired | Intermittent use |
| B4 | 46 | female | otorrhea | 10 | acquired | Intermittent use |
| B5 | 67 | female | otorrhea | 20 | acquired | Intermittent use |
| B6 | 35 | female | otorrhea | 11 | acquired | Intermittent use |
| B7 | 36 | male | otorrhea | 2 | acquired | Intermittent use |
| B8 | 38 | male | hearing loss | 5 | acquired | Intermittent use |
| B9 | 66 | male | otorrhea | 5 | acquired | Intermittent use |
| B10 | 69 | female | otorrhea | 22 | acquired | Intermittent use |
| B11 | 46 | female | otorrhea | 14 | acquired | Intermittent use |
| B12 | 69 | female | earache | 5 | acquired | Intermittent use |
| B13 | 45 | male | otorrhea | 5 | acquired | Intermittent use |
| B14 | 65 | male | otorrhea | 8 | acquired | Intermittent use |
| B15 | 57 | female | otorrhea | 8 | acquired | Intermittent use |
| B16 | 15 | female | otorrhea | 1 | acquired | Intermittent use |
| B17 | 19 | female | earache | 4 | acquired | Intermittent use |
| B18 | 66 | male | otorrhea | 8 | acquired | Intermittent use |
| B19 | 70 | female | hearing loss | 26 | acquired | Intermittent use |
| B20 | 65 | female | otorrhea | 6 | acquired | Intermittent use |
| B21 | 67 | male | otorrhea | 7 | acquired | Intermittent use |
| B22 | 69 | male | otorrhea | 8 | acquired | Intermittent use |
| B23 | 44 | female | otorrhea | 1 | acquired | Intermittent use |
| B24 | 34 | female | otorrhea | 2 | acquired | Intermittent use |
| B25 | 27 | female | earache | 1 | acquired | Intermittent use |
| B26 | 37 | male | otorrhea | 6 | acquired | Intermittent use |
| B27 | 45 | male | otorrhea | 12 | acquired | Intermittent use |
| B28 | 66 | female | otorrhea | 30 | acquired | Intermittent use |
| B29 | 49 | female | otorrhea | 9 | acquired | Intermittent use |
